# Metabolites of non-*aureus* staphylococci affect the ability of *Staphylococcus aureus* to adhere to and internalize into bovine mammary epithelial cells

**DOI:** 10.1186/s13567-023-01232-3

**Published:** 2023-10-26

**Authors:** Bruno Toledo-Silva, Ana Cláudia Dumont Oliveira, Fernando N. Souza, Freddy Haesebrouck, Sarne De Vliegher

**Affiliations:** 1 M-team & Mastitis and Milk Quality Research Unit, Department of Internal Medicine, Reproduction and Population Health, Faculty of Veterinary Medicine, Ghent University, Salisburylaan 133, 9820 Merelbeke, Belgium; 2 Veterinary Clinical Immunology Research Group, Departamento de Clínica Médica, Faculdade de Medicina Veterinária e Zootecnia, Universidade de São Paulo, São Paulo, 05508-270 Brazil; 3 Department of Pathobiology, Pharmacology and Zoological Medicine, Faculty of Veterinary Medicine, Ghent University, Salisburylaan 133, 9820 Merelbeke, Belgium

**Keywords:** Non-*aureus* staphylococci, mastitis, mammary epithelial cell, *S. aureus*, dairy cow

## Abstract

This study investigated whether cell-free supernatants (SN) from four bovine non-*aureus* staphylococcal (NAS) isolates prevent *Staphylococcus aureus* adhesion to and internalization into bovine mammary epithelial cells (MAC-T cells) and if so, to determine whether such effects were potentially associated with the *S. aureus* accessory gene regulator (*agr*) system. Overall, we demonstrated that all SN obtained from the NAS isolates promoted adhesion of a *S. aureus agr*^+^ strain to, yet reduced the internalization into MAC-T cells, while similar effects were not observed for its *agr*^−^ mutant strain. Our findings provide novel anti-virulence strategies for treating and controlling bovine *S. aureus* mastitis.

## Introduction, methods and results

*Staphylococcus aureus* remains a great challenge for bovine udder health in dairy herds, as its treatment and control when causing mastitis often fail due to the complex nature of its virulence factors. The accessory gene regulator (*agr*) locus, a well-known global regulatory system, is a master regulator of *S. aureus* virulence and affects the expression of numerous virulence factors involved in tissue colonization and invasion [1]. Giving the polymorphisms in *agr*B, *agr*C and *agr*D genes of this locus, four *S. aureus agr* specificity groups has been defined [2]—agrI, agrII, agrIII, and *agrIV*—and each of them has been associated with a specific type of mastitis [3]. In this regard, *S. aureus agr* group I is the most prevalent among isolates obtained from bovine subclinical mastitis [3] and is highly associated with intracellular survival in bovine mammary epithelial cells, favoring persistent intramammary infection [1].

We previously demonstrated that metabolites of bovine non-*aureus* staphylococci (NAS) have the capacity to modulate the virulence of a *S. aureus agr*I strain [4]. Therefore, we hypothesize that bovine NAS metabolites also modulate *S. aureus* invasion into bovine mammary gland epithelial cells. Thus, we aimed to evaluate whether supernatants (SN) from four well-studied bovine NAS isolates impact *S. aureus* adhesion to and internalization into bovine mammary epithelial cells and if so, to determine whether such effects may be associated with the *agr* system by comparing an *agr*-positive (*agr*^+^) with an *agr*-negative (*agr*^−^) *S. aureus* strain.

We selected four bovine-associated NAS isolates based on our previous in vitro findings regarding the inhibitory effect of NAS against (methicillin-resistant) *S. aureus* and regarding the effect of NAS SN on the expression of *S. aureus agr*-related genes [4–6]. The *S. chromogenes* IM strain originating from a persistent intramammary infection [7] and the *S. chromogenes* TA strain isolated from a heifer’s teat apex (also referred as “C2” in [8]) were used as well as a *S. simulans* isolate (“SS10”) originating from a teat apex and a *S. epidermidis* isolate (“SE2”) originating from milk. The *S. aureus* strain 8325-4 (*agr*^+^ group I) [9] and its mutant strain 8325-4 Δ*agr* (*agr*^−^) [10], respectively, were included in this study as targets.

The cell-free SN were obtained from the cultures of the NAS isolates, as previously described [4]. Next, the β-galactosidase plate assay was performed to confirm the capacity of NAS SN to regulate the *S. aureus agr*-related virulence genes, with the assessment based on measurement of halo zone diameters (data not shown). As expected, our results substantiated the earlier findings that all NAS SN, except SN from the SE2 isolate, affect the expression of the *S. aureus agr* quorum sensing system [4]. The β-galactosidase plate assay was repeated (data not shown) confirming the previous findings that all NAS SN, except SN from the SE2 isolate, affect the expression of the *S. aureus agr* quorum sensing system [4]. Subsequently, cultures (2 × 10^4^ CFU/mL) of both *S. aureus* strains were washed and resuspended in the different NAS SN. These cultures (NAS SN + *S. aureus*; *n* = 8) were allowed to interact for 2 h prior to challenging bovine mammary epithelial cells with the two *S. aureus* strains, adapted from and modified as follows [11].

A widely used clonal bovine mammary epithelial cell line (MAC-T) was cultured as previously described [12]. After 2 h of interaction between the NAS SN and the respective *S. aureus* strains, the cultures were centrifuged at 3220×*g* for 10 min and the *S. aureus* strains were recovered in MAC-T medium and added to the MAC-T cell culture (2 × 10^4^ cells/well). As positive controls, both *S. aureus* strains without prior contact with NAS SN were also added to the cell culture (1:1 ratio bacteria/cells) and unchallenged MAC-T cells were kept as negative controls. The adhesion and internalization assays were assessed as described by Souza et al. [12]. After 4 h of incubation, the number of bacteria in the MAC-T medium, as well as adhesion and internalization were assessed, and used to calculate the percentage of adhered and internalized bacteria. Each co-culture condition was tested with six replicates.

Statistical analysis were performed using GraphPad Prism 9.4 (GraphPad Software, Inc., San Diego, USA). Adhesion and internalization data were transformed using the squared and z-scores values to obtain normally distributed outcomes variables, respectively. Then, the data were subjected to two-way ANOVA followed by Tukey’s test. It was analyzed whether the percentage of adhered and internalized *S. aureus* to and into MAC-T cells was influenced (1) by SN of all NAS combined as a group and (2) by SN of the four NAS isolates separately, respectively, as compared with the negative controls. The effect of the *S. aureus* strain (*agr*^+^ versus *agr*^−^) was tested as well and the interaction with the NAS variables (NAS group/separate NAS isolates, respectively) was also included. Significance was set at *P* ≤ 0.05. Original median and interquartile range were preserved to improve the interpretation of the results.

**Figure 1:**
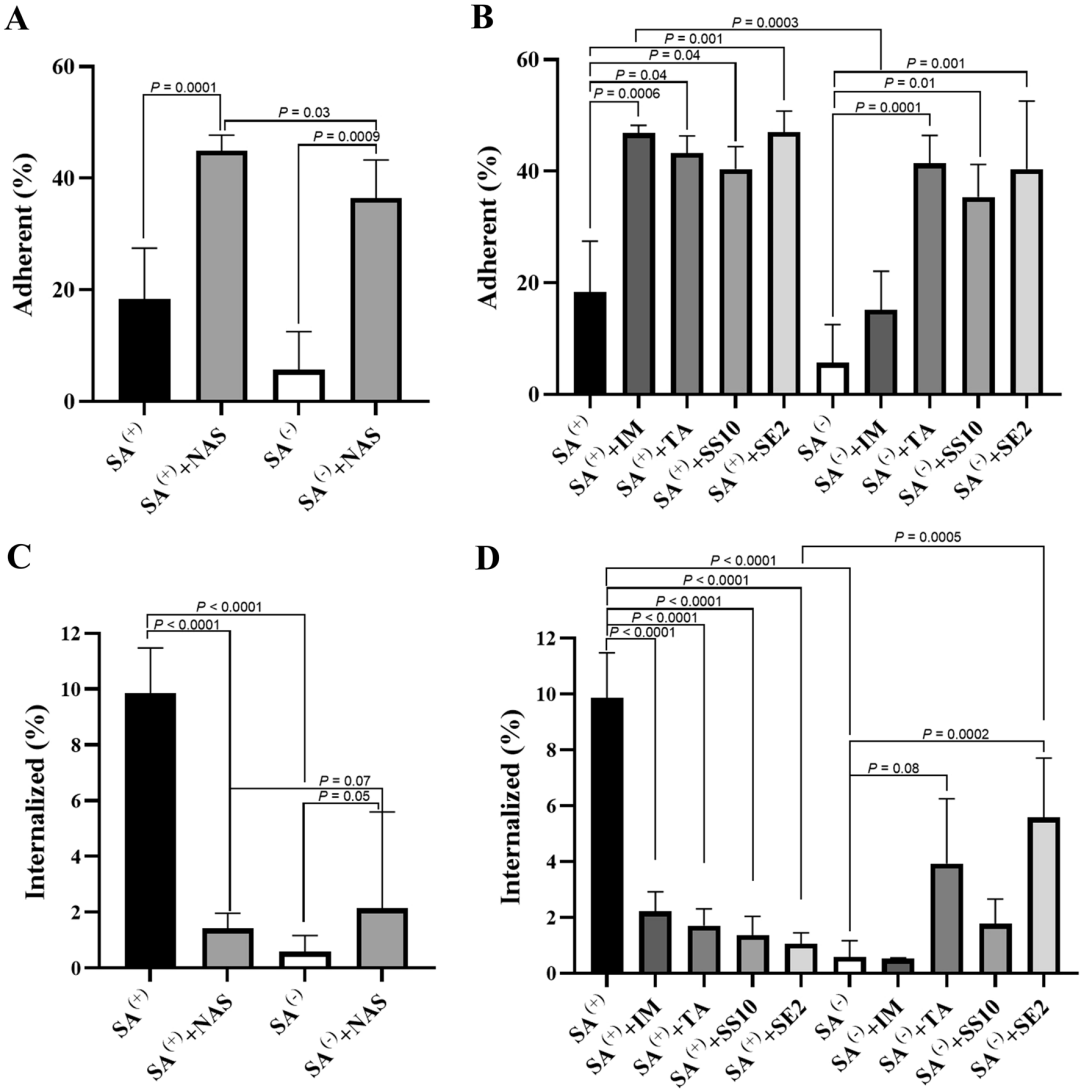
**Non-*aureus*** **staphylococci (NAS) supernatants affect** ***Staphylococcus aureus*** **adhesion to and internalization into MAC-T cells.** Percentage of *Staphylococcus (S.) aureus* strains (*agr*^+^ and *agr*^−^) adherent to (**A** and **B**) and internalized into (**C** and **D**) MAC-T cells with and without previous contact with cell-free supernatants from NAS isolates: *S. chromogenes* IM, *S. chromogenes* TA, *S. simulans* SS10 or *S. epidermidis* SE2. Results are presented as median ± interquartile range.

The adhesion of the *S. aureus agr*^+^ strain onto the bovine mammary epithelial cells did not statistically differ from the *S. aureus agr*^−^ strain (*P =* 0.99; Figure 1A), whereas internalization into the MAC-T cells by the *agr*^+^ strain was more than tenfold higher compared with the *agr*^−^ strain (*P* < 0.0001; Figure 1C).

Prior contact with NAS SN (group) increased substantially *S. aureus* adherence to the bovine mammary epithelial cells (*P* = 0.0001 for the *agr*^+^ strain and *P* = 0.0009 for the *agr*^−^ strain; Figure 1A), an effect that was more outspoken for the *agr*^+^ compared with the *agr*^− ^strain (*P* = 0.03; Figure 1A).

Individually, the SN of all different NAS isolates promoted the adhesion of *S. aureus agr*^+^ strain (Figure 1B), an effect that was also seen for *S. aureus agr*^−^ strain except for the *S. chromogenes* IM SN that did not significantly stimulate adhesion. The significant interaction term between the NAS SN effect and *S. aureus agr* system (*P* = 0.004) indeed underpins the different effects between the different NAS isolates and the two *S. aureus* strains.

Internalization in response to prior interaction with the NAS SN (group) was reduced fivefold for the *S. aureus agr*^+^ strain (*P* < 0.0001) whereas internalization was increased (*P* = 0.05) in the *S. aureus agr*^−^ strain.

All different NAS SN drastically inhibited the internalization of *S. aureus agr*^+^ to a comparable level, while *S. chromogenes* TA (*P* = 0.02) and *S. epidermidis* SE2 (*P* < 0.0001) actually promoted internalization of the *S. aureus agr*^−^ strain (Figure 1D). The significant interaction term between the NAS SN effect and *S. aureus agr* system (*P* < 0.0001) indeed substantiates different effects between the different NAS isolates and the two *S. aureus* strains.

## Discussion

In this study, the capacity of the *S. aureus agr*^+^ strain (*agr* type I) to internalize into bovine MAC-T cells was confirmed, an effect that was almost absent in the *agr*^−^ mutant strain, supporting the hypotheses that the *agr* system is involved in tissue invasion. Previous findings showed that *S. aureus agr* type I, present in the *S. aureus* strain 8325-4 used here, can evade the immune system through internalization into bovine mammary epithelial cells significantly better than isolates carrying other *agr* types [1]. As well, it can persist in the host for a longer period without symptoms of apparent inflammation, also limiting the action of antimicrobials [1], which is very relevant for the control and treatment of bovine *S. aureus* mastitis. The *agr* system is responsible for the coordination of the transition to an invasive mode, which involves increased production of virulence factors and a decrease in surface proteins [13]. Therefore, the inhibition of the *agr* system could be the key to reduce *S. aureus* pathogenicity, antibiotic resistance, and biofilm formation.

In the light of the observations made in the present study, we suggest that the absence of the *agr* regulation, either by the inhibition of *agr* signaling by NAS SN (*S. aureus agr*^+^) or absence of a functional *agr* system (*S. aureus agr*^−^), might be contributing (to some extent) to *S. aureus* adhesion to MAC-T cells. In this scenario, the increased adhesion of *S. aureus* after prior contact with NAS SN to MAC-T cells could potentially lead to a higher recruitment of neutrophils into the mammary gland, which is a well-known pivotal defense mechanism against invading pathogens, as bacterial adhesion to epithelial cells is a strong determinant for the production of neutrophil chemoattractants [14].

A promising finding of our study is that *S. aureus agr*^+^ internalization into MAC-T cells was markedly damped after contact with all NAS SN (Figure 1C), which is important for the pathogenesis. Interestingly, the udder-adapted *S. chromogenes* IM strain was able not just to reduce the internalization of *S. aureus agr*^+^, but also to hinder *S. aureus agr*^−^ internalization into the MAC-T cells (Figure 1D). These findings provide further evidence on the putative protective effect of the *S. chromogenes* IM strain in relation to bovine udder health [4, 5, 15–17].

Another intriguing finding is that *S. aureus agr*^−^ internalization into MAC-T cells increased after prior contact with SN from *S. chromogenes* TA and *S. epidermidis* SE2 (Figure 1D). Surprisingly, previous findings of our research group showed that the same NAS isolates were capable to promote biofilm formation of the *S. aureus agr*^−^ strain and to suppress biofilm dispersion of the same *S. aureus* strain [5]. Altogether, our results indicate that certain metabolites produced and secreted by NAS isolates, which are also present in the SN, may have the ability to influence the internalization of *S. aureus* via a mechanism unrelated to the *agr* system.

On one hand, the inactivation of the *agr* system results indeed in a greater potential for cell invasion [18]. On the other hand, the inhibition of *agr* signaling in *S. aureus agr*^+^ strain by NAS SN most probably triggered a shift of the expression of exoproteins such as *spa* [4, 18] that decreases cell internalization capacity. However, intrinsic factors from both *S. aureus* strains and SN from NAS isolates may have potentially contributed to the differences in the degrees of internalization observed in our study. Consistent with the unique behavior exhibited by NAS strains, prior research has revealed significant variations in virulence [19], potential beneficial properties [4, 5, 8, 16], and host-interaction [12, 20, 21] among and within bovine-associated NAS species. Hence, further studies are required to explore and identify metabolites produced and secreted by distinct NAS, their nature of action, and their potential use for combating infections, including bovine intramammary infection.

We conclude that the mechanism behind the dichotomous behavior of *S. aureus* adhesion to and internalization into bovine mammary epithelial cells by NAS SN is NAS isolate*-*dependent, and this effect is likely reliant on the *S. aureus agr* system although we cannot dismiss the potential involvement of unmeasured *S. aureus* traits and particular NAS metabolites. Still, as internalization of *S. aureus* into bovine mammary epithelial cells plays a key role in immune evasion [12] and that bacterial adherence to epithelial cells is as a strong determinant for neutrophil chemoattractant production [14], our findings open new perspectives to explore innovative anti-virulence strategies targeting treatment and control of bovine *S. aureus* mastitis.

## Data Availability

The manuscript provides the data upon which the conclusions of the manuscript are based.
